# Antibiofilm activity of terbinafine, itraconazole and their combination against *Sporothrix brasiliensis* yeast-like form’s biofilms

**DOI:** 10.1007/s42770-026-01869-0

**Published:** 2026-04-27

**Authors:** Anarosa B. Sprenger, Terezinha I. E. Svidzinski, Marlon R. Geraldo, Andreia B. Silva, Leticia A. M. Sant Ana, Regielly C. R. Cognialli, Emanuel Razzolini, Germana D. Santos, Vania A. Vicente, Flavio Queiroz-Telles

**Affiliations:** 1 Internal Medicine Graduate Program, Federal University of Parana, Curitiba, Paraná Brazil; 2 Microbiology, Parasitology and Pathology Graduate Program, Federal University of Paraná, Curitiba, Paraná Brazil; 3 Visiting professor at the Basic Pathology Department, Federal University of Paraná, Curitiba, Paraná Brazil; 4 Hospital de Clínicas, Federal University of Paraná, Curitiba, Paraná Brazil; 5 Bioprocess and Biotechnology Engineering Graduate Program, Federal University of Parana, Curitiba, Paraná Brazil

**Keywords:** Antifungal susceptibility, Sporotrichosis, Yeast biofilms, Antifungal agents. Drug resistance, Antibiofilm drugs, Combination therapy

## Abstract

*Sporothrix brasiliensis* biofilms represent a challenge in the treatment of sporotrichosis due to their structural complexity and antifungal resistance. This study evaluated the efficacy of terbinafine (TERB) and itraconazole (ITZ), and their synergism against biofilms of yeast-like forms of *S. brasiliensis*. Seven clinical strains were analyzed for biofilm formation and structural alterations using spectrophotometry, confocal laser scanning microscopy (CLSM), and scanning electron microscopy (SEM). The treatment in early-stage biofilms (3 hours) exhibited greater susceptibility to ITZ, whereas mature biofilms (144 hours) responded better to TERB. Statistical analysis confirmed significant differences among treatments (*p* < 0.016) and a dose-dependent effect, although no considerable synergistic interaction was observed between ITZ and TERB. CLSM and SEM analyses revealed reduced biofilm formation and adhesion, biofilm disorganization, and cellular morphological changes following exposure to antifungals, both individually and in combination. TERB was significantly more effective in eradicating mature biofilms than ITZ or the combination (*p* < 0.001). These findings reinforce the need for early intervention in sporotrichosis treatment and suggest that TERB may be a valuable alternative for biofilm-associated infections.

## Introduction

Sporotrichosis is an implantation mycosis caused by fungi of the *Sporothrix* genus, with *Sporothrix brasiliensis* being the primary species associated with epi-zoonotic transmission in Brazil [1]. This thermodimorphic fungus grows as filaments at 25–28°C and as yeast at 36–38°C, which is its parasitic form in human and other vertebrate animals [2]. Sporotrichosis presents as either localized skin lesions or, in immunocompromised individuals, severe disseminated disease [3].

Standard treatment for this disease involves antifungals such as itraconazole, terbinafine, and amphotericin B; but some cases do not respond even after extended therapy [4]. Oral terbinafine has demonstrated efficacy in the treatment of several systemic mycoses, including sporotrichosis, with a favorable safety profile, and has shown clinical benefit when combined with itraconazole in refractory cases [5–10]. The combination of itraconazole and terbinafine has already demonstrated efficacy and safety in some clinical studies of sporotrichosis, as well as in other systemic mycoses [6, 11, 12].

In recent years, the role of *S. brasiliensis* biofilm formation and its potential contribution to antifungal resistance has gained attention [13, 14]. Biofilms are structured microbial communities adhered to biotic (alive, human cells) or abiotic surfaces (medical devices) [15–17].

This fungal architecture is encased in a protective extracellular matrix. The biofilm event provides microorganisms with enhanced protection against the host immune system and chemical agents [18]. Consequently, biofilm formation may increase the antifungal resistance and its impact on therapeutic efficacy. *In vitro* studies on *S. brasiliensis* biofilm formation and antifungal susceptibility have been conducted, most of them have focused on the filamentous phase of this fungus [19], which may exhibit greater antifungal resistance, as revealed in a recent study conducted by do Prado et al. (2025) [20].

In this context, the present study investigates the antibiofilm activity of itraconazole and terbinafine, as well as the interaction between them against *S. brasiliensis* biofilms, specifically under the yeast-like phase.

## Materials and methods

### Strains study

A total of seven human *Sporothrix brasiliensis* clinical strains (Table 1) were obtained from patients with forms of cutaneous-lymphatic sporotrichosis treated at the infectious disease outpatient clinic from the Hospital de Clínicas of the Federal University of Paraná (UFPR), Curitiba, Brazil.

**Table 1:** *Sporothrix brasiliensis* reference strains used in this study

Collection Identification Number/Genbank acsses number	Source
CMRP 3967/OQ571295	Lymphocutaneous abscess
CMRP 5491/In process	Conjunctival secretion
CMRP 2525/MG869808	Peritoneal fluid
CMRP 3955/OQ571286	Right forearm biopsy
CMRP 5318/ON397982	Right arm abscess
CMRP 3944/In process	Skin biopsy
CMRP 5083/ON397983	Secretion from wrist lesion

*CMRP* Microbiological Collections of Paraná Network

The strains were identified and stored in Sabouraud dextrose agar (KASVI Ltda). All the strains were deposited in the Microbiological Collections of Paraná Network-Taxonline - (https://www.cmrp-taxonline.com). Initially two successive cultivations in brain heart infusion (BHI) agar at 37 °C for five days were performed for thermoconversion from the filamentous form to the yeast-like form [21]. Throughout the experiments, the strains were kept under these conditions. The yeast forms of the strains were confirmed by lactophenol cotton blue staining, which revealed only cigar-shaped yeast cells and no septate hyphae, indicating full conversion to the yeast phase [22, 23].

### Antifungal drugs

Two antifungals were used in this study: itraconazole (ITZ; Sigma-Aldrich, Burlington, MA, USA) and terbinafine (TERB; Sigma-Aldrich, Burlington, MA, USA). The drugs were diluted in dimethyl sulfoxide (DMSO) as recommended in the document M27-A3 of the Clinical and Laboratory Standards Institute (CLSI). The solutions were stored at −25°C.

### Biofilm Formation and Development

The inoculum for biofilm production was prepared in 0.15 M of phosphate-buffered saline, pH 7.4 (PBS), according to the methodology used by Brilhante et al (2017) [24] in a study on the in vitro susceptibility of biofilms of the *S. schenkii* complex. The inoculum concentration was measured in a Neubauer chamber, and the cell concentration adjusted to 1.75–9.30 x 10^6^ CFU/mL, to each strain evaluated in this study.

For the growth kinetics and biomass determination, a total of six 96-well microplates were filled with 100 μL of each isolate inoculum suspension and 100 μL of RPMI medium. The assays were performed in duplicate and twice repeated.

### Antifungal susceptibility of *S. brasiliensis* biofilms

#### Antifungal solutions for treatment

For the biofilm assays, stocks solutions of TERB (50 μg/mL) and ITZ (400 μg/mL) both in DMSO, were prepared to achieve final four times the MICs concentration determined previously (data no shown). Both solutions were diluted 10 times, whose final concentration ranged from 0.002 to 1 μg/mL for TERB and 0.016 to 8 μg/mL for ITZ. The microplates for evaluating drug interactions were prepared according to the method described above, but before assays, above solutions were mixed so that the 10 concentrations ranged from 0.004/0.03μg/mL to 0.125/1 μg/mL with TERB and ITZ respectively.

#### Biofilm inhibition assay

After inoculum preparation, 1000 μL aliquots of RPMI solution were added to four 96-well plates containing round polystyrene coverslips (SPL Life Sciences Co., South Korea) The plates were sealed and incubated at 37 °C under orbital shaking at 120 rpm for seven days. After 3 hours of incubation, plates with biofilm prepared were washed twice with PBS solution. RPMI solutions containing TERB, ITZ and mix solution of both drugs (TERB/ITZ) at the concentrations detailed above were added the different plates. The negative control (OD _blank_) consisted of 200 μL of uninoculated RPMI medium added to the initial wells on the left. For the positive controls, an inoculum ranging from 1.75 to 9.30 x 10^6^ CFU/mL in RPMI medium without drug was introduced into the final wells on the right (OD _control_).

#### Biofilm eradication test

The remaining three plates were also washed after 3 hours of incubation, so 200 μL of RPMI (without antifungal drugs) were added. All plates were sealed and incubated at 37 °C, at a rotation of 120 rpm, until 144 hours. After that, the plates that had received only RPMI were washed and treated in the same manner as the inhibition and were designated as "mature biofilm". The antifungal drugs evaluated (single and combination solutions) were added on plates with mature biofilms. All plates remained in the shaker incubator under the same temperature and rotation conditions for an additional 24 hours.

#### Analysis of antibiofilm activity

Quantitative antibiofilm activity of the antifungals (TERB, ITZ, and TERB/ITZ) was evaluated through the determination of total biomass, and the morphological and structural assessment of biofilm behavior through confocal microscopy (CLSM) and scanning electron microscopy (SEM) assays.

#### Total biomass determination

Biomass quantification was performed using crystal violet staining, based on dye retention by fungal biomass and measured by spectrophotometry. In the crystal violet assay, after 24 hours of drug treatment, wells were washed three times with PBS to remove non-adherent cells. Subsequently, fungal structures were fixed with 99% methanol. Then, the methanol was removed, and 0.3% filtered crystal violet solution was added. After 30 minutes of incubation at 35 °C, the dye was removed, and wells were washed again twice with 200 μL of PBS. Afterward, 150 μL of 33% acetic acid was added to each stained well and left for 30 seconds. Finally, the contents of the wells were transferred to another microplate and immediately read in a spectrophotometer at 540 nm which measured the absorbance for crystal violet stain providing values of optical density (OD) [24–27].

#### Determination of biofilm inhibitory concentration (MBIC)

Through the analysis of optical density results, the minimal biofilm inhibitory concentration (MBIC) was recorded as the lowest concentration of antifungals or that inhibit 100% of biofilm, was calculated using the formula below:

$$\%\textrm{Biofilm}\ \textrm{inhibition}\ \textrm{activity}=\frac{\left(\textrm{OD}\ \textrm{control}\hbox{--} \textrm{OD}\ \textrm{blank}\right)\hbox{--} \left(\textrm{OD}\ \textrm{test}\hbox{--} \textrm{OD}\ \textrm{blank}\right)}{\textrm{OD}\ \textrm{control}\hbox{--} \textrm{OD}\ \textrm{blank}}\times 100$$

#### Determination of biofilm eradication concentration (MBEC)

The determination of the lowest concentration of antifungals (TERB, ITZ, and TERB/ITZ) capable of reducing 100% of mature biofilm was calculated using the percentage of biofilm eradication, whose formula is represented below [28]:

$$\%\textrm{Biofilm}\ \textrm{eradicating}\ \textrm{activity}=\frac{\left(\textrm{OD}\ \textrm{control}\hbox{--} \textrm{OD}\ \textrm{blank}\right)\hbox{--} \left(\textrm{OD}\ \textrm{test}\hbox{--} \textrm{OD}\ \textrm{blank}\right)}{\textrm{OD}\ \textrm{control}\hbox{--} \textrm{OD}\ \textrm{blank}}\times 100$$

### Statistical analysis

The statistical analysis performed using SPSS 20, with descriptive measures such as mean, standard deviation, minimum, and maximum calculated for each concentration level. Line graphs were created to visually assess the behavior of the variables across concentrations. To observe the impact of concentrations on optical density, a Generalized Linear Mixed Model (GLMM) was applied, regarding fixed effects (concentration) and random effects (variation between samples). The significance of the effects was assessed with *p* < 0.05. The optical density values in relation to increasing doses of TERB, ITZ, and TERB/ITZ were showed by the graphs using the Prism 8.0.1 program.

### Confocal Laser Scanning Microscopy (CLSM)

After incubation, all plates were washed twice with PBS, stained with LIVE/DEAD fluorescent dye (Invitrogen, USA), and examined using a Nikon ECLIPSE Ti-E microscope. Live cells were detected at 488 nm with SYTO 9, while dead or damaged cells were detected at 561 nm using propidium iodide [29–31]. Images were acquired in the x-y plane at 1 μm z-intervals, and biofilm reconstructions were created with Fiji-Image J software.

### Scanning Electron Microscopy (SEM)

The morphology and structural biofilms changes caused by antifungals was observed by SEM. The biofilms formed were fixed onto polystyrene coverslips by adding 1 mL of Karnovsky's solution for 24 hours. After this period, the Karnovsky's solution was aspirated, and the wells were washed three times with cacodylate buffer (0.05 M, pH 7.4). Five minutes later, the coverslips were dehydrated twice for 10 minutes each with increasing ethanol concentrations (30%, 50%, 70%, 80%, 90%, and 99,5%). Finally, the plates were taken immersed in 99.5% alcohol to the Electron Microscopy Center/UFPR, where samples underwent critical point drying, and the slides were mounted on the sample holder using conductive tape, followed by gold sputter-coating. Image acquisition was performed using a TESCAN MIRA3 scanning electron microscope [32–34].

## Results

### Ability of biofilm formation by *Sporothrixbrasiliensis* yeast-like forms

**Fig. 1:**
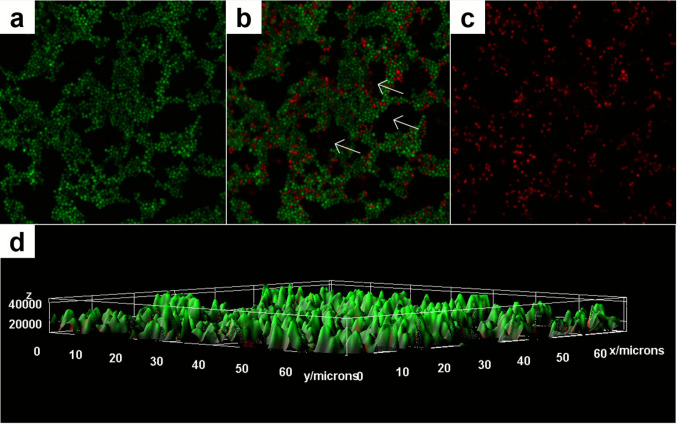
Confocal laser scanning microscopy (CLSM) of *Sporothrix brasiliensis* mature biofilm without antifungal treatment stained by SYTO9 and propidium bromide. (**a**) Dense, compact biofilm composed of architecturally organized, viable rounded cells (green); (**b**) The same biofilm showing a small proportion of non-viable cells (red) with evident cell-free spaces (white arrow); and (**c**) Sparse rounded non-viable cells (red). Magnification: 400x; (**d**) 3D reconstruction by Confocal Laser Scanning Microscopy (CLSM) of *Sporothrix brasiliensis* without antifungal treatment

The biofilm formation capacity of yeast-like forms of *Sporothrix brasiliensis* was evaluated through CLSM and SEM. The CLSM revealed dense and compact biofilms, composed mainly of round cells stained green (live) by SYTO9, cell-free spaces interspersed in the biofilm framework, indicating non-viable (dead) cells stained red by propidium bromide (Fig. 1). The three-dimensional (3D) reconstruction (Fig. 1d) showed control biofilms with a thickness of 4000 μm, displaying a robust and compact structure, predominantly composed of live cells (green).

**Fig. 2:**
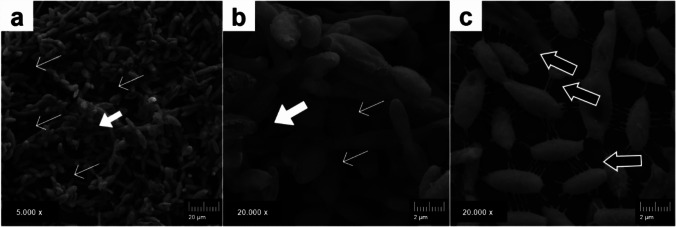
Scanning electron microscopy (SEM) of yeast-like *Sporothrix brasiliensis* CMRP 2525 mature biofilm. (**a**) Biofilm formed by cigar-shaped cells interconnected, with extracellular matrix (wide white arrows) and water channels (narrow white arrows); (**b**) 20.000x magnification of panel (**a**), showing cigar-shaped cells clustered, with extracellular matrix (wide white arrows) and water channels (narrow white arrows); (**c**) Yeast-like *S. brasiliensis* cigar-shaped cells, some of them connected, with intercellular extensions between them (black arrows), in a greater magnification (20.000 x), some cells showed cellular extensions

In the SEM, biofilms were composed of cigar-shaped cells connected by an extracellular matrix containing openings (water channels), and at higher magnification, some cells displayed cellular extensions between them (Fig. 2).

### Evaluation of antifungals antibiofilm activity

The initial approach used to evaluate the antibiofilm activity of itraconazole and terbinafine, alone and in combination, was based on optical density measurements obtained by spectrophotometry experiments to determine the minimal biofilm inhibitory concentration (MBIC) and minimal biofilm eradication concentration (MBEC). These parameters were calculated *for Sporothrix brasiliensis* strains, as described in the Materials and Methods section. The results of these assays are presented in Table 2.

**Table 2:** MBIC and MBEC results for different *Sporothrix brasiliensis* strains treated with Itraconazole (ITZ), Terbinafine (TERB), and their combination (TERB/ITZ)

Strains	Antifungals (μg/mL) MBIC/MBEC		
	ITZ	TERB	TERB/ITZ
CMRP 2525	0.03/0.03	0.004/0.004	0.002–0.017/0.002–0.017
CMRP 5491	0.03/0.063	0.002/0.002	0.002–0.018/0.125–1.125
CMRP 5083	0.016/0.016	0.002/0.004	0.002–0.017/0.016–0.125
CMRP 5318	0.016/0.016	0.002/0.016	1–8/>1/8
CMRP 3944	0.03/0.016	0.002/0.008	0.125–1/0.063–0.5
CMRP 3967	0.03/0.016	0.004/0.008	0.25–2.2/0.002–0.016
CMRP 3955	0.013/0.063	0.002/0.063	0.002–0.017/0.002–0.016

*MBIC* minimal biofilm inhibitory concentration, *MBEC* lowest concentration of antifungals that reduce 100% of biofilm, *ITZ* itraconazole, *TERB* terbinafine.

**Fig. 3:**
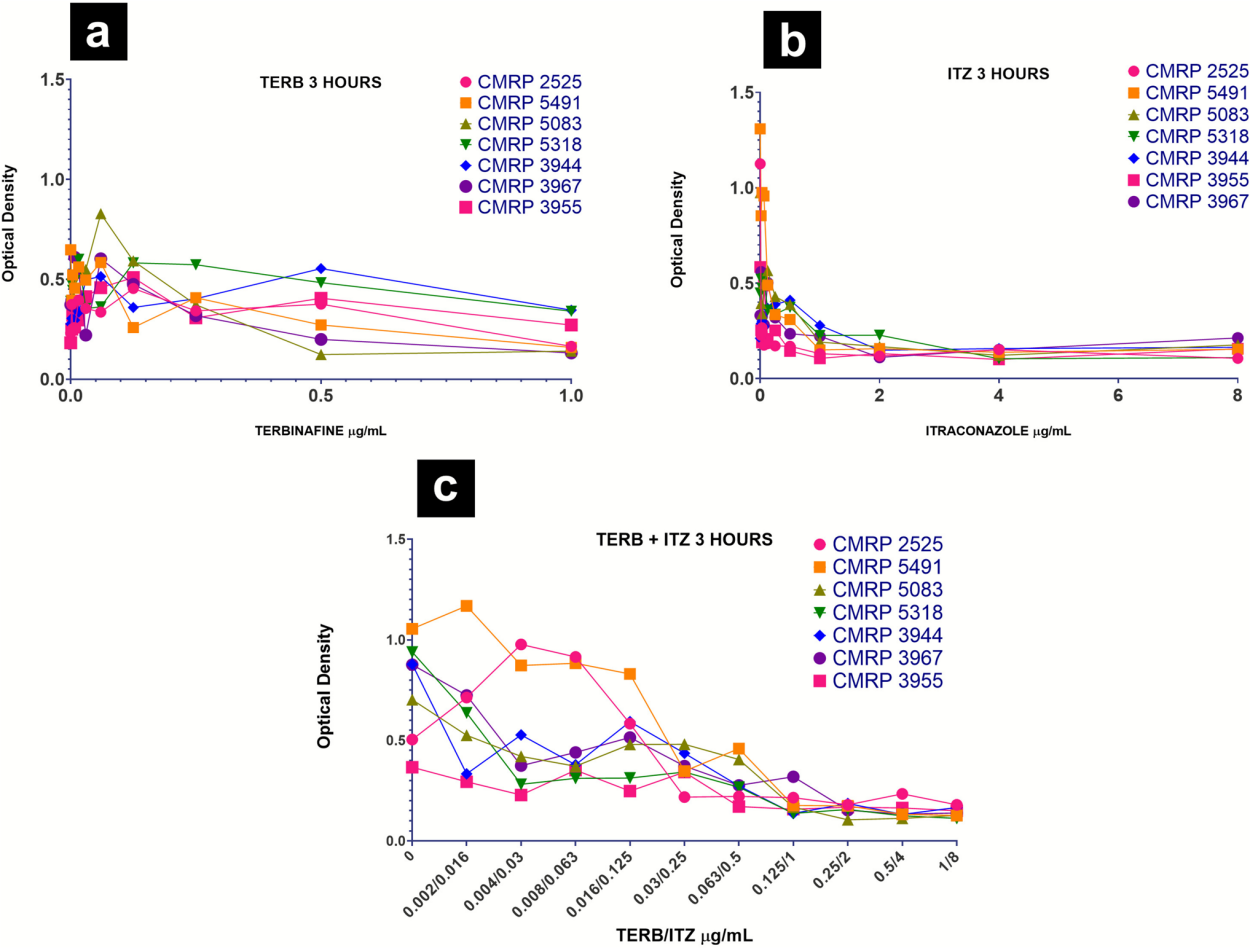
Variation of inhibition of *Sporothrix brasiliensis* biofilms, after addition of increasing concentrations of (**a**) TERB, (**b**) ITZ and (**c**) TERB/ITZ after adherence period

Biofilm formation inhibition was evaluated through optical density by spectrophotometry of the inhibition plates for determination of MBIC. The analysis of the optical density in relation to the increasing concentrations of TERB, ITZ and TERB/ITZ added at 3-hour of incubation (after adherence time) demonstrates the beginning of the decrease in optical density from 0.5 μg/mL for TERB, 1 μg/mL for ITZ and 0.125/1 μg/mL for TERB/ITZ (Fig. 3).

**Fig. 4:**
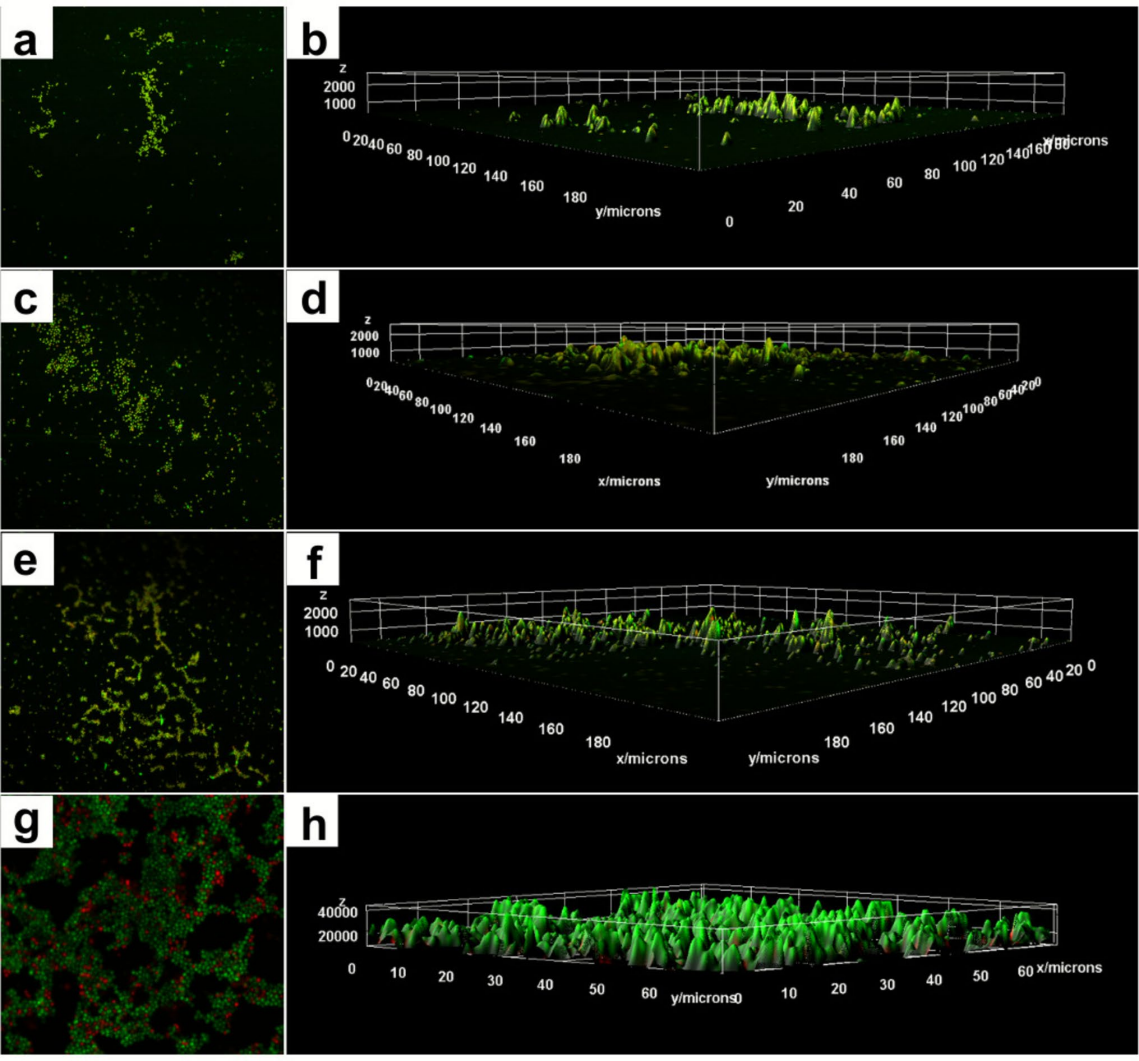
Confocal laser scanning microscopy (CLSM) illustrating the effect of early antifungal treatment *Sporothrix brasiliensis* CMRP 5491 biofilms compared with untreated control biofilms. (**a**-**b**) TERB (0.5 μg/mL), (c-d) ITZ (1 μg/mL), (**e**-**f**) combination of TERB/ITZ (0.125/1 μg/mL), Magnification 200 x.; (**b**), (**d**) and (**f**): 3D image of panels (**a**), (**c**) and (**e**); (**g**): Dense, compact biofilm composed of architecturally organized control biolfim without treatment; (**h**) 3 D image of panel (**g**)

Confocal microscopy of biofilms treated with 0.5 μg/mL of TERB, 1 μg/mL of ITZ, and the combination TERB/ITZ at 0.125/1 μg/mL after 3 hours of incubation revealed a reduced number of rounded cells, lacking defined architectural organization, mostly stained green (live), mixed with a few red-stained cells (dead). (Fig. 4).

The three-dimensional MCVL revealed, in addition to the presence of a smaller quantity of stained structures, a smaller thickness of the biofilms (close to 1000 μm) compared to the thickness of the control biofilm (4000 μm). (Fig. 4 b, d and f).

**Fig. 5:**
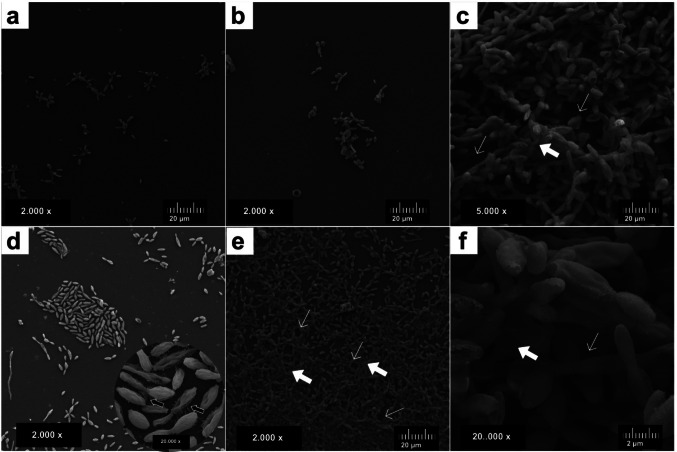
Scanning electron microscopy (SEM) images illustrating the effect of early antifungal treatment (3 hours of incubation) on Sporothrix brasiliensis CMRP 5491 biofilms, compared with untreated control biofilms. (**a**) TERB (0.5 μg/mL) and (**b**) ITZ (1 μg/mL) (2,000×); (**c**) Untreated biofilm treatment showing greater number of adhered cells, extracellular matrix (wide white arrow) and water chanels (narrow white arrows). (5.000x); (**d**) combination (TERB 0.125/ITZ 1 μg/mL) (2.000x), the detailed image revealed altered cell morphology and reduced intercellular fibrils (black arrows) (20,000×); (**e**) The lower-dose combination (TERB 0.004/ITZ 0.03 μg/mL) (2.000×); (**f**) Untreated biofilm showing cigar-shaped cells clustered, with extracellular matrix and water channels

The SEM analysis of biofilms treated with higher antifungal concentrations showed isolated or small groups of cigar-shaped cells. At higher magnification, clear alterations in cell morphology and a reduction in connecting fibrils were observed. On the other hand, SEM of biofilms treated with lower concentrations of antifungals revealed well-formed, compact biofilms with evident extracellular matrix and water channels (Fig. 5).

#### Mature biofilms behavior after antifungal addition

**Fig. 6:**
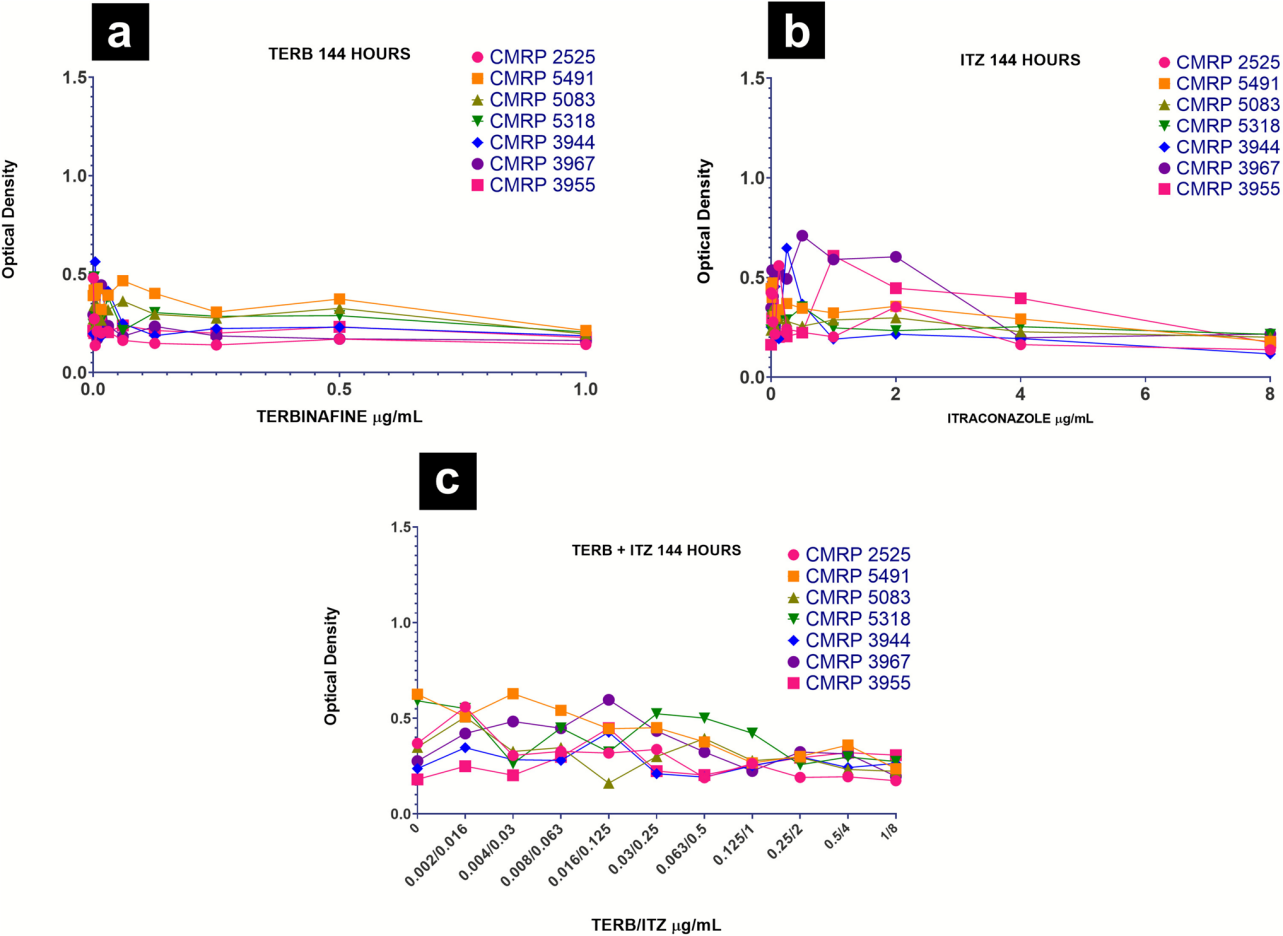
Variation in mature biofilms of *Sporothrix brasiliensis* under different antifungal concentrations. (**a**) TERB; (**b**) ITZ and (**c**) TERB/ITZ

In mature biofilms with drug added after 144 hours of incubation, it was observed a the decrease in optical density from 0.5 μg/mL for TERB, 2 μg/mL for ITZ and 0.125/1 μg/mL for TERB/ITZ with a trend towards stability and homogeneity at lower optical densities up to higher concentrations for all strains (Fig. 6).

**Fig. 7:**
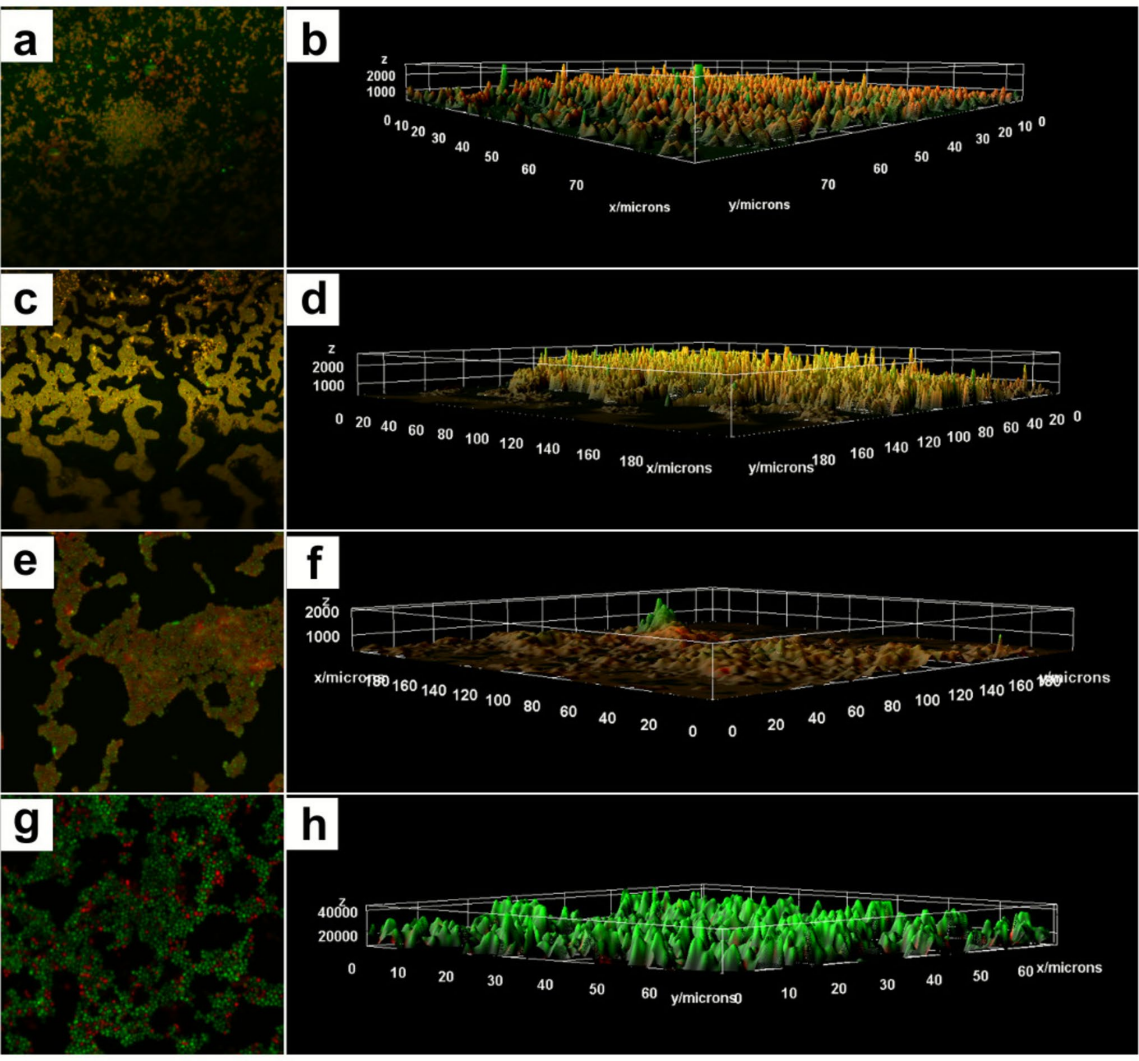
Confocal laser scanning microscopy (CLSM) of *Sporothrix brasiliensis* CMRP 5491 mature biofilms after treatment with antifungals compared with untreated control biofilms. (**a**-**b**) TERB (0.5 μg/mL), (**c**-**d**) ITZ (1 μg/mL), (**e**-**f**) combination of TERB/ITZ (0.125/1 μg/mL) - Magnification: 400x.; (**b**), (**d**) and (**f**): 3D image of panels (**a**), (**c**) and (**e**), with (**b**), Magnification: 400 x.; (**g**): Untreated control biofilm; (**h**) 3D image of panel (**g**)

In addition, the CLSM, showed mature biofilms containing greater cells surface compared to those treated at 3 hours. The overall biofilm architecture was preserved even at the highest antifungal concentrations; however, a significant increase in the number of non-viable cells (red-stained) was observed. The overlap of green- and red-stained cells in the same regions resulted in a more orange-toned appearance of some biofilms. The 3D estriutures observed by the CLSM showed that despite the preserved architecture, the mature biofilms presented reduced thickness (below 2000 μm) after treatment with antifungals, compared to the thickness of mature biofilms without treatment (4000 μm), in addition to the presence of larger areas stained red or even the overlap of red over green, making the color of some biofilm’s orange. (Fig. 7).

**Fig. 8:**
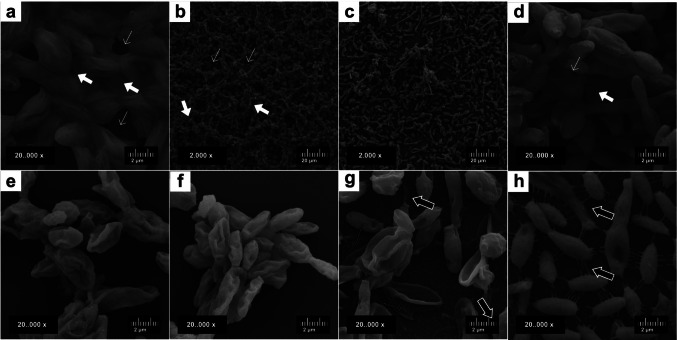
Scanning electron microscopy of *Sporothrix brasiliensis* CMRP 5491 mature biofilms after antifungal treatment compared with untreated control biofilms. (**a**) and (**e**) TERB (0.5 μg/mL); (**b**) and(**f**) ITZ (1 μg/mL); (**c** and **g**) Combination of TERB (0.125 μg/mL) and ITZ (1 μg/mL); (**d**) and (**h**) untreated mature biofilm, showing no alterations in the shape of cells or fibers. Thick white arrows: dense extracellular matrix; thin white arrows: water channels; Black arrows:intercellular fibers; Dashed white arrows: disrupted fungal cell

Scanning electron microscopy of mature biofilms demonstrated preservation of their architecture, characterised by a dense extracellular matrix and discernible water channels (Fig. 8 a-b). Biofilms exposed to higher concentrations of the antifungal combination terbinafine (0.5 μg/mL) and itraconazole (1 μg/mL) exhibited marked structural disorganisation, with diminished matrix density (Fig. 8 c and f). At increased magnifications, notable morphological changes in fungal cells were evident across all biofilms, including reduced connecting fibrils and disruption of the fungal cell wall (Fig. 8 d, e, and f).

### Statistical analysis

The statistical analysis indicated a significant difference among antifungal treatments regarding inhibition of *S. brasiliensis* biofilms (*p* = 0.016) and a significant interaction between antifungal and concentration (*p* < 0.001), revealing a dose-dependent effect. At concentrations ITZ 0.03 to 0.25 μg/mL and TERB/ITZ 0.004/0.03 to 0.03/0.25 μg/mL, itraconazole and the antifungal combination showed significantly lower optical density compared to terbinafine (*p* < 0.05). However, Itraconazole exhibited higher MBIC values, suggesting that higher concentrations were required to inhibit early biofilm formation. Terbinafine displayed a more variable MBIC, indicating a less consistent response among the evaluated strains. The antifungal combination did not demonstrate a clear *in vitro* advantage over assays using only itraconazole.

For mature biofilms, MBEC values differed significantly between treatments (*p* < 0.001), while no significant interaction was found between antifungal type and concentration (*p* = 0.846), indicating similar effects across doses. Comparative analysis showed that terbinafine was significantly more effective than both itraconazole and the antifungal combination in reducing mature biofilms (*p* = 0.004 and *p* < 0.001, respectively). Itraconazole exhibited higher MBEC values, reinforcing that higher doses were required for eradication. The antifungal combination showed no clear benefit over assays using only Itraconazole.

## Discussion

Fungal biofilm formation is a complex process driven by quorum sensing, enabling cell and molecular interactions in the host environment [35] The results confirmed the ability of the yeast-like forms of *Sporothrix brasiliensis* to form organized and structured biofilms in their yeast morphology, as previously reported in other studies [36–38]. The inhibition of biofilm formation was evaluated by introducing antifungal agents three hours after incubation, a period representing initial cellular adhesion, according already described [25, 36, 38].

Variability in antifungal tolerance was observed among the strains. Strains CMRP 3955 and CMRP 5491 exhibited the highest MBEC values for itraconazole, suggesting greater structural resilience to this antifungal agent. For terbinafine, strain CMRP 3955 again stood out as the most resistant, requiring higher concentrations for biofilm eradication. In the ITZ/TERB combination, strain CMRP 5318 showed the most resistant profile, with maximal MBIC/MBEC values markedly higher than those of the other isolates. This behavior may be related to intra-species genetic and phenotypic heterogeneity and the ability of certain strains to modulate antifungal tolerance in the biofilm state, as previously reported in susceptibility studies of *S. brasiliensis* and *S. schenckii* biofilms [20, 24].

The methodological approach applied in this study – standardized biofilm induction, antifungal exposure during adhesion and maturation phases, and structural analysis by CLSM and SEM - aligns with protocols previously validated for other pathogenic fungi. Brilhante et al. (2017) [24] evaluated antifungal susceptibility of filamentous-phase biofilms of *Sporothrix schenckii* using similar conditions.

More recently, Brilhante et al. (2023) [39] and Costa et al. (2025) [40] applied comparable methodologies to analyse the *Histoplasma capsulatum* biofilms exposed to itraconazole, amphotericin B, and chitosan-based formulations. Together, these studies support the reproducibility and applicability to other species of the methodological pattern employed in this study.

The ultrastructural patterns observed in this study are in line with damage profiles previously described in fungal biofilms exposed to antifungals, although detailed reports specifically focusing on *Sporothrix* biofilms remain scarce [16]. Itraconazole had the efficacy described against biofilms of several fungal species in the literature, including *S. schenckii* sensu stricto, *S. globosa*, *Candida parapsilosis*, *C. albicans, A. fumigatus Histoplasma capsulatum* [15, 17, 24, 31, 33, 38, 41]

Although the number of studies evaluating the antibiofilm activity of terbinafine are more limited, the available evidence indicates its efficacy against biofilms of dermatophytes such as *Trichophyton rubrum* and *T. interdigitalis*, as well as against non-dermatophyte fungi such as *Scopulariopsis brevicaulis*. Conversely, terbinafine shows lower effectiveness against *Candida albicans* biofilms [17, 42].

The six-day incubation period perfomed was sufficient for the development of structurally organized biofilms that may hinder antifungal activity. This finding reinforces the importance of early diagnosis and treatment of sporotrichosis, as according to the literature [43, 44], patients generally seek medical assistance only after several days or weeks of infection progression. In clinical practice, the delay therapeutic can compromise efficacy, particularly due to the formation of biofilms [3, 45–47].

Corroborating previous studies, this work demonstrated relative resistance of *S. brasiliensis* biofilms to itraconazole (ITZ) and terbinafine (TERB). Quantitative biomass analysis showed greater ITZ activity in inhibiting initial biofilms, whereas TERB was more effective against mature biofilms [48, 49].

According our results, MBIC values indicated that higher ITZ concentrations were necessary to inhibit biofilms, while TERB, despite requiring lower inhibitory concentrations, showed variable responses among strains.

The distinct performance of ITZ and TERB during the adhesion and maturation phases may be attributed to the mechanisms of action of each antifungal. Itraconazole, being a triazole, inhibits ergosterol biosynthesis, compromising the cell membrane integrity, which may explain its greater efficacy in the initial biofilm phase, with limited structural organization [15, 50]. The greater efficacy of terbinafine in biofilm eradication can be related to its inhibition of squalene epoxidase [16, 51], which not only blocks ergosterol biosynthesis but leads also to the accumulation of squalene, a fungitoxic compound [9]. In addition, its lipophilic nature facilitates diffusion through the extracellular biofilm matrix, known to contain some lipid components, as well as the lipid chains of cell membranes [8, 20]. Terbinafine lipophilicity facilitates tissue distribution, which may explain its capacity to reach compacted areas such as fibrotic regions and granulomas, which is particularly relevant in deep and chronic fungal infections [12, 35, 50, 52–57].

Despite the morphological changes observed by CLSM and SEM after combined treatment with ITZ and TERB, no statistically significant synergistic effect was detected in the inhibition or eradication of biofilms, in contrast to reports suggesting synergistic interaction for other *Sporothrix* species or in clinical cases involving mucosal infections [11, 58, 59]. This lack of synergism may be related to the absence of complementary mechanisms of action or to a possible antagonistic effect on antifungal penetration through the biofilm matrix [12].

In addition, phenotypic alterations provide fungal cells organized in a biofilm with living conditions that are quite different from their planktonic counterparts. One of the most significant differences is the well-documented resistance of biofilms to antifungal agents [35]. The matrix can impede the access of antifungals to the cells embedded within the biofilm, supposedly by decreasing the rate of transport or by binding the antifungals extracellularly, thus hindering their penetration into the cells [41]. This attribute has been described in other human pathogenic fungi such as *Candida albicans*, *Aspergillus fumigatus* [60], and *Cryptococcus neoformans* [61]. The mechanism of fungal biofilm formation and regulation is closely related to the external environment but also involves a series of complex genes and regulatory pathways. For example, in *Saccharomyces cerevisiae*, the *Flo*11 gene is important for biofilm formation and increased resistance to amphotericin B [62].

Although *in vitro* studies such as this indicate greater efficacy of terbinafine against mature biofilms, itraconazole remains the first-line therapy for sporotrichosis due to pharmacokinetic factors, bioavailability, and interactions with the host immune system [63–68]. Thus, the results of this study showed new insights demonstrating that yeast-like forms of *S. brasiliensis* form structured biofilms within a few hours, resistant to antifungal activity. The evaluation of the biofilm response to exposure to itraconazole and terbinafine, alone or in combination, showed morphological and structural changes, but no evident antifungal synergism was evalueted. These findings reinforce the need for further studies aimed at confirming these results and providing new inferences, for example, regarding assays based on planktonic forms, which will be important to guide diagnostic and therapeutic strategies in the clinical management of sporotrichosis, considering the complexity and resistance observed in *in vitro* studies.

This study expands the understanding of antifungal responses in adherent structures, particularly regarding the behavior of *S. brasiliensis* biofilms when exposed to the TERB/ITZ combination, which, to our knowledge, had not yet been described in the literature.

## Data Availability

The datasets generated and analyzed during the current study, including raw data, spreadsheets, and supplementary materials, are available from the corresponding author upon reasonable request, in: https://drive.google.com/drive/folders/164G4QKaO43wk3tCeLy-8prGqXg7aEYXk?usp=sharing

## Abbreviations

CFU: Colony-forming units
CHC-PR: Clinical Hospital Complex of Paraná
CLSI: Clinical and Laboratory Standards Institute
CLSM: Confocal laser scanning microscopy
CMRP: Microbiological Collections of Paraná Network
CV: Crystal violet
DMSO: Dimethyl sulfoxide
GLMM: Generalized Linear Mixed Model
ITZ: Itraconazole
MBEC: Minimum biofilm eradication concentration
MBIC: Minimum biofilm inhibitory concentration
MCVL: Microscopy for Confocal Visualization of Live cells
OD: Optical density
PBS: Phosphate-buffered saline
RPMI: Roswell Park Memorial Institute medium
SEM: Scanning electron microscopy
TERB: Terbinafine
UFPR: Federal University of Paraná
